# Effective University Facility Management Plan Proposal Reflecting the Needs of the Main Users

**DOI:** 10.3389/fpsyg.2020.00219

**Published:** 2020-02-18

**Authors:** Min Soo Kim, Jun Ha Kim

**Affiliations:** Department of Housing and Interior Design, Kyung Hee University, Seoul, South Korea

**Keywords:** university facility management, UFM items, UFM strategies, importance–performance analysis, multiple linear regression analysis

## Abstract

As the low birthrates in Korea intensify, the average school-age population is showing great reduction and the resources used for admission to the university are gradually decreasing as well, due to the continuing economic downturn. Therefore, in order for the university to remain competitive and keep up with the fast paced changes, it is very significant that they induce qualitative growth of university education and facilities through extensive and thorough analysis on the university facility management (UFM) for students who are the main users of university facilities. However, research done on university facilities in Korea has been focused mainly on space management, while the effective UFM reflecting in-depth opinions on the user class has been quite inadequate. Therefore, in order to improve student satisfaction and improve the efficiency of UFM, it is crucial to understand the importance and status of the UFM items. This way, an initial plan for improvement of UFM considering the priorities that actively reflect the opinions of the students is prepared in this research. For the UFM items, the eight classifications of UFM, which can be evaluated by users, and the details of the classifications are used for this research. For the UFM strategies, the first 176 performance indicators (PIs) are collected, consolidated, and deleted. Finally, eight UFM strategies are derived. In order to find out which UFM items need more focus on, importance–performance analysis (IPA) is conducted. The priority of management is determined by where each factor is located on the grid. Additionally, multiple linear regression analysis is conducted to examine the effect of the importance value on the UFM items on the importance value on the UFM strategies. Finally, the average values of importance for the strategies of UFM of two groups are compared and analyzed. As a result of the stages listed above, this research attempts to provide basic data on preparing the UFM plan. Therefore, it is possible to apply the method that reflects the needs of actual users in preparing future UFM plans throughout the research methods proposed in this research.

## Introduction

Universities are the institutions that play a central role when it comes to driving technological development and social changes as the infrastructure for performing various functions such as education, research, and community service. As we enter this era, the role of universities and social responsibility to foster the level of human resources required by society along with national competitiveness are expected to increase (Kim et al., 2006; Hassanain, 2008; Akinyode, 2014).

Despite the importance of the various roles that universities hold, most Korean universities are currently faced with a serious crisis (Jeon, 2009). As the low birthrates in Korea intensify, the average school-age population is showing great reduction and the resources used for admission to the university are gradually decreasing as well, due to the continuing economic downturn. In fact, in 2015, the number of universities across the country was 390, including 51 national and public universities, and 339 private universities. However, the number of colleges and universities decreased to 384, with 49 national and public universities and 335 private universities in 2018. As a result, not only is competition among domestic universities intensifying, but additionally global competition among universities are showing greater rivalry, and efforts to secure qualitative competitiveness of university education and facilities are becoming a major required factor.

Therefore, in order for the university to remain competitive and keep up with the fast paced changes that will be occurring in the future, it is very significant that they induce qualitative growth of university education and facilities through extensive and thorough analysis on operation and management for students who are the main users of university facilities (Reynolds and Cain, 2006; Kim et al., 2018).

However, research done on university facilities in Korea has been focused mainly on space management, while the effective university facilities management reflecting in-depth opinions on the user class has been quite inadequate. Unlike other countries, in Korea, the research that is performed based on the factors that influence the learning outcomes of university facilities are not active, which leads to the need of users in the field to obtain higher education (Shin and Kim, 2012). Under all of these circumstances, applying facility management (FM) strategy to the university facilities is a strong requirement (Shin and Kim, 2012). FM in universities generally mean that it provides a suitable environment for education and research purposes which is the main objective of the university. By operating and maintaining the university facilities in an optimal state, it reduces the operating costs by optimizing maintenance activities. In other words, university facilities, unlike general facilities, consist of facilities that require diverse functions, such as basic education facilities, research facilities, and support facilities. The need for research on efficient management is even higher.

**FIGURE 1:**
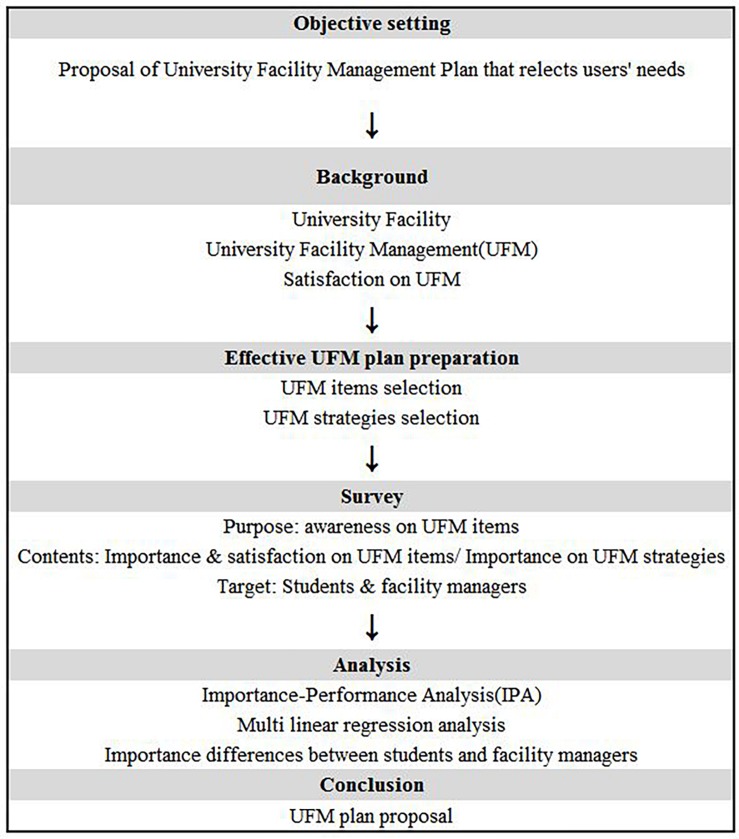
Research flow.

Therefore, in order to improve student satisfaction and improve the efficiency of university FM (UFM), it is crucial to understand the importance and status of the UFM items. This way, a plan for improvement of UFM considering the priorities that actively reflect the opinions of the students can be prepared in this research (Figure 1).

## Background

### University Facility

University facilities are the basic hardware when it comes to university research, education, and academic activities (Lee and Cho, 2008). It is also a spatial background in which various functions, such as university’s main education, research, and student’s rest, are performed. It has already been verified through many previous studies that these university facilities, just like other educational facilities, perform a great influence on the quality of education provided in the university and the satisfaction of students as the main users (Kim et al., 2006; Hassanain, 2008; You and Lee, 2008). Association of higher educational facilities in the United States indicates that universities can effectively achieve their predetermined goals and objective by the systematic FM performance measure (Appa, 2007). University facilities are largely divided into four facilities: basic education facilities, support facilities, research facilities, and attached facilities according to Article 4 of the Korean College Operational Regulations (Table 1). These university facilities are composed of various functions such as educational function, research function, and support function. The facility can tend to be complex and used by various users, so it follows with various requirements for maintenance. Accordingly, in order for efficient management to take place, it is necessary to prepare an effective management plan in consideration of the users’ position.

**TABLE 1:** Classification of university facilities.

**Classification**	**Division**
Educational basic facilities	Lecture room, laboratory, faculty laboratory, administration room, library, student center, university headquarters and its facilities. The library should have the following facilities (1 and 2)
	1. Reading room, periodical room, reference book reading room, library, and office
	2. Seats in the reading room that can accommodate >20% of the student capacity
Support facilities	It is composed of gymnasium, auditorium, electronic computing center, training factory, student dormitory, and its facilities.
Research facilities	It is a laboratory for research, a graduate school, a research institute affiliated with a university and its auxiliary facilities.
Attached facilities	Houses or apartments of museums, faculty, staff, graduate students, and researchers. And their auxiliary facilities and affiliated schools

### University Facility Management

The International FM Association (IFMA) defines FM as a profession that encompasses multiple disciplines to ensure functionality, comfort, safety, and efficiency of the built environment by integrating people, place, process, and technology. Another definition comes from the 29 European countries, where they defined FM as an integration of processes within an organization to maintain and develop the agreed services which support and improve the effectiveness of its primary activities (EN15221-1: 2006 FM – part 1: terms and conditions). UFM is an integrated service activity that is comprehensive and needs long-term planning and management for the purpose of minimizing costs, maximizing utility, and flexible maintenance. This can be done by optimizing fixed assets that are used and held for university purposes. For example, physical services can be done in order to ensure that a building’s air conditioning is operating effectively or safely. In depth, physical services are a series of actions to confirm whether a building’s heating and cooling are operating effectively or safely (Kim, 2016).

Moving on, intangible services refer to a service that checks whether a building is kept clean and is well supervised by a contractor or a manager. FM is frequently used similarly for space management or asset management, however, FM can tend to be limited when it comes to commercial assets that are a bit more complex to manage and operate or have more of a broader spatial scope.

Furthermore, the university’s FM is directly related to the financial aspects, and the facility maintenance management routinely inspects, maintains, and repairs damaged facilities so that they can preserve the function of the completed facility and also enhance the convenience and safety of users. The legal definition of facility maintenance management is focused on corrective maintenance in the comprehensive FM domain. Yet, the term “FM” in this research covers the academic content of FM, which is beyond its legal meaning. Additionally, other studies pointed out that the necessity for preparing and making systematic, active, and effective future FM plans (Shon et al., 2003; Cho and Lee, 2008; Ryu and Lee, 2008; Yun et al., 2009). Therefore, in this research, “FM” is referring to the above-mentioned corrective maintenance in reactive manner and also the wide ranging activities performed for the efficient use of the facility done by effectively planning and managing that prevents future accidents or inefficiencies.

### Satisfaction on UFM

The satisfaction research on the educational facility has been actively conducted all around the world (Kim, 2019). One factor that all of these studies have in common is that satisfaction is measured from a variety of perspectives. Astin (1993) categorized the limited satisfaction level by estimating the students “satisfaction in a specific field, such as surveys of students” perceptions, the professor’s lecture scores, and administrative services. Yorke et al. (2000), on the other hand, provides satisfaction to the United Kingdom university students by labeling them by the areas of curriculum organization, teaching and learning, library, computer training facilities, computer facilities, student service, school environment, restaurants and lounges, student council activities, and self-development opportunities. Ruben (1995) classified satisfaction as quality of teaching, quality of administrative service, and quality of teaching and learning. Lastly, LeBlanc and Nguyen (1999) evaluated satisfaction by analyzing it into functional value, epistemological value, social reputation, justice value, economic value, and social value.

## Materials and Methods

### UFM Items Selection

This research conducted a literature review of Korean Standard (KS) and previous studies to establish a classification of UFM items that can be evaluated by users. Our research tools are based on the previous research (Shin et al., 2015) that adopted “FM KS FM Service Standard Specification (KS S 1004-1: 2011)” and the “KS FM Standard Specification Based on KS FM Standard Specification.” The eight classifications of UFM, which can be evaluated by users, and the details of the classifications are used for this research. The total number of users consists of 57 male (39.6%) and 87 female college students. The classification and details of FM survey tools used are shown in Table 2. There are eight classifications of university facilities management survey tools set up in this study, starting with “Ensuring the Safety of the Facility” and seven more areas.

**TABLE 2:** Classification and items of UFM.

**Classification**	**Details**	**Satisfaction (*A*)**	**Importance (*B*)**	**Gap (*B*−*A*)**
		**Mean**	**Mean**	
Ensuring the safety of the facility	1. Eliminate and manage traffic obstacles to ensure the safety of pedestrian walkways	3.43	4.16	0.73
	2. Secure and manage spaces for evacuation such as lecture halls in case of emergency	3.15	4.22	1.07
	3. Ensuring and managing enough spaces for traffic and emergency vehicles (fire trucks, ambulance, etc.)	3.13	4.29	1.15
	4. Always secure spaces for evacuation around lecture building in case of emergency	3.2	4.27	1.07
Crime prevention of facility	5. Establish integrated control system and emergency contact system for police and security companies to cope with crime	3.22	4.17	0.95
	6. Management of pedestrians to ensure visibility to prevent crime and crime anxiety	3.37	3.88	0.51
	7. Installation and management security equipment such as emergency bell and CCTV to prevent crime and crime anxiety	3.36	4.23	0.87
	8. Installation and management of security authentication device that controls night access at the entrance of building	3.52	4.04	0.52
Facility inspection and repair	9. Secure facility performance standards and management manuals	3.16	3.93	0.77
	10. Dedicated management team for quick repair and replacement after deterioration of facility	3.09	3.94	0.85
	11. Record management to prevent recurrence and prompt response	3.14	3.9	0.75
	12. Continuously maintain facility performance to flexibly respond to changes in facility use and demand	3.29	3.97	0.67
Maintain indoor environment	13. Obtain and manage efficient energy usage data by analyzing usage patterns	3.06	3.97	0.9
	14. Installation and management of individual facilities that can operation air conditioning and heating equipment	3.31	4.08	0.76
	15. Remote management system installation for efficient monitoring and control of energy consumption in all seasons	3.26	3.76	0.49
	16. Maintain indoor air temperature control system for environment-friendly facility	3.31	4.07	0.75
	17. Maintain indoor air humidity control system for environment-friendly facility	3.21	3.98	0.77
Public facilities management	18. Obstacle removal management for versatility and easy access to pedestrian walkways and small spaces	3.5	3.94	0.44
	19. Maintain facilities for the use of guidance system, temperature, and humidity for elevator users	3.37	3.83	0.45
	20. Maintain support facility for voice and braille guidance in university facilities for the disabled	3.02	4.11	1.09
	21. Maintain small space equipment for students’ learning and rest	3.12	3.9	0.77
	22. Provide manuals to maintain and manage the quality and performance of lockers for students’ personal storage	3.19	3.9	0.7
Maintenance and management of equipment	23. Manage spaces so that the quantity, model, and use can be changed according to the purpose of lecture room use	3.23	3.8	0.56
	24. Manage spaces to store necessary equipment according to the purpose of lecture room use	3.17	3.75	0.58
	25. Maintain facilities to support various learning spaces such as lectures and information exchange	3.36	4.07	0.71
	26. Periodic replacement and management to maintain the quality and performance of the finishing materials	3.19	3.87	0.67
	27. Manage fixtures and storage spaces according to the number of people	3.11	3.71	0.6
Lighting environment	28. Provide and manage artificial lighting environment to provide suitable environment for lectures and learning	3.32	3.92	0.6
	29. Provide and manage artificial lighting environment to provide suitable exchange and rest	3.19	3.83	0.63
	30. Install and manage user-sensing sensors and automatic extinguishing systems to reduce energy usage	3.14	3.765	0.62
	31. Install and manage windows for natural light control to adjust the brightness of the indoor spaces and improve the efficiency of lighting	3.11	3.73	0.61
Sanitation and water supply	32. Secure and manage the proper space and number of the toilet for the convenience	3.55	4.15	0.6
	33. Provide and manage water quality that meets legal standards when installing water supply facilities	3.41	4.13	0.72
	34. Manage toilet air conditioning equipment to maintain a comfortable and convenient environment	3.34	4.21	0.87
	35. Manage clean and sage entry and exit for easy and safe access to restrooms	3.56	4.28	0.72
	Overall mean	3.26	3.99	0.73

### UFM Strategies Selection

In order to derive the strategy of UFM, performance indicator (PI) data are collected. In-depth analysis of previous researches used to identify management tasks carried out in universities. Using this data, previous research data related to domestic and foreign PIs are investigated and analyzed.

First, 13 papers and conference proceedings related to FM service evaluation index are collected. After that procedure, PIs of basic UFM services are analyzed, and then PIs for domestic and foreign university facilities are analyzed. In order to narrow down the scope of UFM strategies to these collected PIs, PIs with high similarity are consolidated, followed by two Delphi surveys of UFM experts (Table 3).

**TABLE 3:** Results of the 1st Delphi survey and eight UFM strategies.

**Number**	**Performance indicators (PIs)**	**Mean**
1	Public expenses	4.45
2	Building maintenance costs	4.36
3	Operation costs	4.27
4	Estimated maintenance costs	4.00
5	Service expenses	3.64
6	Assessment of adequacy of facility security	4.20
7	Assessment of adequacy of space allocation	4.13
8	Customer satisfaction measurement activity	3.87
9	Safety management activity	4.47
10	Energy target management activity	4.20
11	Water consumption management activity	4.00
12	Security management activity	4.00
13	Management basic plan establishment activity	4.00
14	Space management activity	4.00
15	Preparation of space management regulations	3.87
16	Preparation of computer-aided FM system	3.80
17	Establishment of space usage schedule activity	3.67
18	Manpower acquisition and management activity	4.36
19	Employee satisfaction evaluation activity	4.07
20	Development activity through training program	4.07
Overall average (out of 5.0)		4.07

**FIGURE 2:**
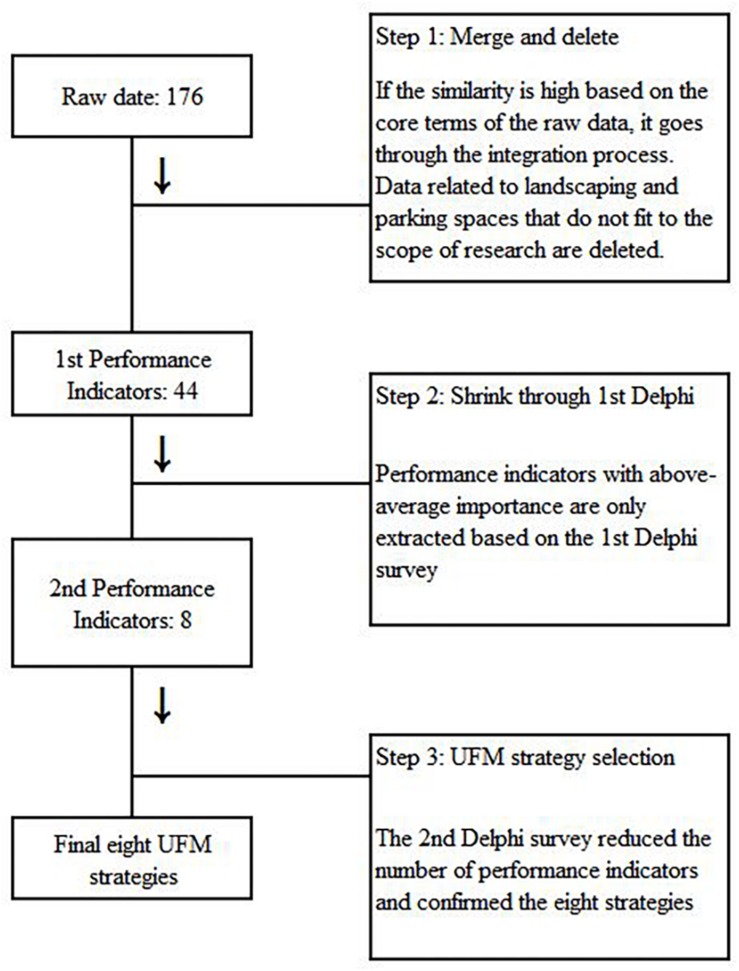
UFM strategy determination process.

Determining the necessity of integrating and modifying the PIs, which are the raw data, in order for finally selecting the strategy of the UFM, the first 176 PIs are collected/consolidated and deleted. Finally, eight UFM strategies are derived (Figure 2).

A total of 176 pieces of original data of PIs related to UFM are collected, excluding items related to the exterior of the building, parking, site, landscaping, construction, moving, etc., which are out of scope in this research. For a case of high similarity, 44 PIs are determined through the integration process.

An expert group consisted of UFM practitioners at Korean universities were asked to investigate the importance of the extracted PIs, Delphi survey. A panel of experts was selected under one condition; at least over 5 years professional experiences in a UFM. Thirty-three UFM experts (23 male and 10 female) participated to evaluate the importance of PIs and this survey is conducted using the five-point Likert scale. The data collected in the first and second rounds are analyzed using SPSS 20. Based on the mean value of the importance, 20 PIs scored above average are extracted. Subsequently, an additional second Delphi survey is conducted to select final UFM strategies based on the importance of the PIs (Table 3). According to a three-point scale (“1” = not necessary, “2” = important but not essential, “3” = essential), the content validity ratio (CVR) is also applied to ensure the adequacy of eight UFM strategies and the equation 1 was used as follows:

(1) C⁢V⁢R=(n-N/2)/(N/2)

where *n* is the number of experts indicating a UFM strategy is “three-essential” and *N* is the total number of experts on the panel. All eight UFM strategies got a CVR of 0.33 or higher indicating that at least 66% of the panel (22 out of 33 experts) rates the items to be essential. There are three lagging PIs (#1, 2, and 3) that are output oriented, relatively easy to measure but hard to improve, on the other hand, there are five leading PIs (#6, 7, 9, 10, and 18) that are input oriented, relatively easy to improve but hard to measure accurately.

### Importance–Performance Analysis

In order to consider the relationship between two different factors such as the importance and the performance, importance–performance analysis (IPA) method is used (Martilla and James, 1977). This research is to examine the average value through satisfaction and importance survey on 35 university facilities management items, and then to select the items to input management manpower and cost through IPA. As shown in Table 2, the users’ overall satisfaction level is 3.26 and the importance level is 3.99, indicating that Gap (*B*−*A*) 0.73 shows a lower level of satisfaction compared to the importance of overall management items.

**FIGURE 3:**
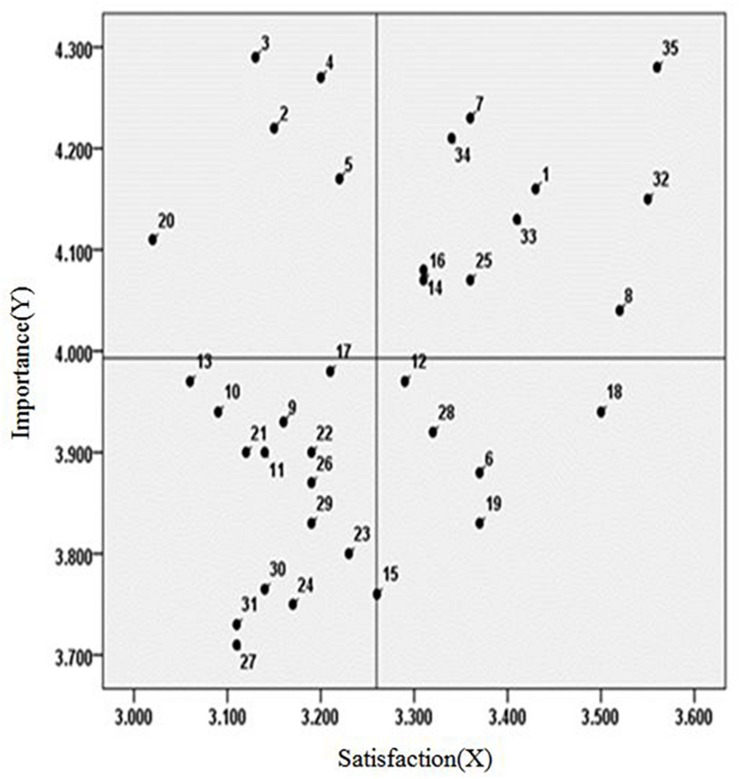
Importance–performance analysis on UFM items.

Therefore, as shown in Figure 3, it is fundamental to adjust the direction toward improvement of the satisfaction of the five management items in the second quadrant. This can be done by adjusting and redistributing resource inputs to the five management items in the fourth quadrant. In detail, the items with low satisfaction to importance, five items that need to be improved by urgently inputting human and material resources are “secure passage space” (#3), “voice and braille guide (#20),” ensuring evacuation space in side of the building “(#2),” ensuring evacuation space around the building “(#4),” establishing a control system “(#5).” On the other hand, the five items with high satisfaction to importance that have an excessive input of human and material resources are “obstacle removal management for various activities” (#18), “maintenance of guidance systems” (#19), “securing field of vision” (#6), “continuous maintenance of facility performance” (#12) and “providing artificial lighting environment” (#28).

## Results

### Multiple Linear Regression Analysis of UFM Items for UFM Strategies

In order to identify the UFM items that affect the strategy of UFM, multiple linear regression analyses are conducted in order to find out how an UFM item is numerically related to the UFM strategy, examining the effect of the importance value on the UFM items on the importance value on the UFM strategies (Table 5). The tolerance limit of all variables are >0.1 and the VIF value is <10, so it is judged that there is no problem on the collinearity. The overall explanatory power (*R*^2^) of the regression equation is 15.9–36.8%. The following table shows the UFM items that affects the eight UFM strategies (Table 4).

**TABLE 4:** Multi-linear regression of UFM items for UFM strategies.

**Dependent variables**	**Independent variables**	**Non-standardized coefficient**		**Standardized coefficient**	***t***	**Significance**	**Collinearity statistics**	
		***B***	**SE**	**β**			**Tolerance**	**VIF**
Operating expenses-related activities	(Constant)	2.289	0.332		6.896	0		
	Water quality management	0.234	0.084	0.266	2.773	0.007	0.773	1.294
	Artificial lighting management	0.195	0.071	0.261	2.725	0.007	0.773	1.294
	*R*^2^: 0.205							
Bldg. O&M-related activities	(Constant)	2.045	0.385		5.313	0		
	Facility performance standard and manual	0.278	0.091	0.290	3.052	0.003	0.803	1.245
	Water quality management	0.205	0.088	0.222	2.335	0.021	0.803	1.245
	*R*^2^: 0.190							
Public expenses-related activity	(Constant)	1.219	0.418		2.916	0.004		
	Temperature control system	0.206	0.099	0.208	2.087	0.039	0.664	1.506
	Security authentication system	0.211	0.083	0.229	2.533	0.013	0.808	1.238
	Individual equipment for heating/cooling	0.226	0.094	0.227	2.403	0.018	0.743	1.346
	*R*^2^: 0.266							
Facility acquisition adequacy	(Constant)	1.835	0.475		3.863	0		
	Finishing material quality control	0.265	0.09	0.275	2.945	0.004	0.775	1.291
	Water quality management	0.284	0.101	0.274	2.801	0.006	0.709	1.411
	Integrated control system	−0.216	0.088	−0.229	2.446	0.016	0.774	1.292
	Individual equipment for heating/cooling	0.206	0.094	0.197	2.185	0.031	0.83	1.205
	*R*^2^: 0.256							
Space allocation adequacy	(Constant)	2.266	0.321		7.055	0		
	Finishing material quality control	0.448	0.081	0.462	5.532	0	1	1
	*R*^2^: 0.213							
Energy goal management	(Constant)	2.159	0.414		5.215	0		
	Finishing material quality control	0.26	0.084	0.282	3.08	0.003	0.894	1.118
	Individual equipment for heating/cooling	0.204	0.091	0.205	2.236	0.027	0.894	1.118
	*R*^2^: 0.159							
Safety management	(Constant)	1.911	0.408		4.684	0		
	Securing evacuation space	0.294	0.105	0.286	2.805	0.006	0.553	1.809
	Water quality management	0.335	0.093	0.32	3.586	0.001	0.72	1.389
	Maintenance for rest	−0.237	0.076	−0.266	3.124	0.002	0.796	1.257
	Security equipment installation	0.193	0.095	0.213	2.034	0.044	0.525	1.907
	*R*^2^: 0.368							
Manpower procurement	(Constant)	1.981	0.406		4.884	0		
	Sensor detection and automatic light off management	0.251	0.085	0.268	2.95	0.004	0.893	1.119
	Security equipment installation	0.232	0.086	0.244	2.689	0.008	0.893	1.119
	*R*^2^: 0.174							

Among the entire UFM items, the items that affect the “operation cost-related activities” of UFM and have high standardized coefficient beta are “water quality management (0.266)” and “artificial lighting management (0.261).” This is related to energy such as water resources and electric energy, and it can be seen that students think there is a high correlation between energy consumption and operating cost. In connection with the entire UFM items, the items that alter the “building O&M-related activities” and have a high standardized coefficient beta are “securing facility performance standards manual (0.290),” “water quality management (0.222).” Through this, it can be concluded that college students believe that building O&M-related activities cost can be reduced by management plans, for instance, securing management manuals and strengthening water quality management.

The items that influence the “public expenses” and have high standardized coefficient beta are “temperature control system (0.208),” “security authentication system (0.229),” and “individual equipment for heating and cooling operation (0.227).” It shows us that students tend to think that temperature control systems and heating and cooling operation systems can possibly reduce the public expenses by controlling the usage. With the introduction of security systems, the public expenses are expected to be scaled down by saving the input of human resources. The items that are involved with the change of the “facility acquisition adequacy” and have high standardized coefficient beta are “finishing material quality control (0.275),” “water quality management (0.274),” “integrated control system (−0.229),” and “individual equipment for heating and cooling” (0.197). A possible conclusion from this result, as the contents of securing the facility itself are about the interior space where students mainly live, is that it is related to quality control of finishing materials. Another thing that can be spotted is that that the heating and cooling control determines the appropriateness of securing the indoor space.

The items that affect the “space allocation adequacy” and have high standardized coefficient beta are “finishing material quality (0.462).” Considering that the main spaces used by students are lecture halls and rest spaces, the use of appropriate finishing materials can be used as a criterion for judging the suitability of students for space allocation. The items that modify the “energy goal management” and have high standardized coefficient beta are “finishing material quality (0.282)” and “individual equipment for heating and cooling.” To sum it up, the operating equipment of heating and cooling machinery, which is directly related to energy, influences the energy goal management. Likewise, the correlation between the quality control of the interior finishing material and the energy consumption is immense. The items that affect the “safety management” and have high standardized coefficient beta are “securing evacuation space (0.286),” “water quality management (0.320),” “maintenance for rest (−0.266),” and “security equipment installation (0.213).” Safety management is one of the most significant aspects of UFM strategies and it can be seen that the management of crime prevention and security system can make a difference when discussing the matter of safety for our students. Securing the evacuation space is also another factor that is directly related to their safety in case of an emergency. There is a negative correlation with the management items related to the rest space and as shown, the management items related to rest are not associated with the psychological stability of students. The last items that affect the “manpower procurement” and have high standardized coefficient beta are “sensor detection and automatic light off management (0.268)” and “security equipment installation (0.244).” Regarding securing manpower, students seem to think that manpower resources can be diminished by using automatic extinguishing systems and security equipment.

In conclusion, UFM items that influence the eight UFM strategies the most are “water quality management” (that has the positive impact on the four strategies), “operating expenses-related activities,” “bldg. O&M-related activities,” “facility acquisition adequacy,” and “safety management.” Based on these results, in order to prioritize UFM items in the future, it is mandatory to manage the water quality from a higher level as well as managing cost-related items, facilities, and safety. The followings are the UFM items that commonly have an effect on the strategy of UFM (Table 5).

**TABLE 5:** UFM items commonly affecting UFM strategies.

**Number**	**UFM items**	**Frequency**	**Multi-relational UFM strategies**
1	Water quality management	4	Operating expenses-related activity, Bldg. O&M-related activity, facility acquisition adequacy, safety management
2	Individual equipment for heating/cooling	3	Public expenses-related activity, facility acquisition adequacy, energy goal management
3	Finishing material quality control	3	Facility acquisition adequacy, space allocation adequacy, energy goal management
4	Security equipment installation	2	Safety management, manpower procurement

### Importance Differences in the Strategy of UFM Between Users and Managers

It is commonly assumed that the problem of UFM is not being operated efficiently. However, issues are caused when FM is conducted without accurately reflecting the needs of users of university facilities. Therefore, this research examines the difference in the strategy of UFM between users and facility managers (Table 6). As a result of examining the importance gap between users and facility managers, all areas show negative values. In general, facility managers tend to guess that the strategy of UFM is more critical than students who use facilities. The biggest difference among them is “public cost-related activities (electricity, gas, and water resource usage fees).” This indicates that for facility managers, cost reduction is much more important than for students. In reality, it can be seen that they respond more sensitively toward the costs incurred in operating the facility. The second contrast among them is “manpower procurement.” In other words, it means that it is more of a priority to the manager who directly manages it, than the students who use the facility. On the other hand, the item with the least difference is shown as “safety management.” This is the most important FM goal in both groups along with both groups identifying safety as their top priority. Among the three items related to costs, there are differences that are higher than the average cost in “building O&M-related activities” and “public expenses-related activities.” This indicates that managers see the cost reduction as the main strategy of FM compared to users who value convenience in simply using facilities.

**TABLE 6:** Importance differences between students and facility managers.

**Number**	**UFM strategies**	**Students’ importance (*A*)**	**Managers’ importance (*A*)**	**Gap (*B*−*A*)**
1	Operating expenses-related activities	4.02	4.27	−0.25
2	Bldg. O&M-related activities	3.98	4.36	−0.38
3	Public expenses-related activities	3.83	4.45	−0.62
4	Facility acquisition adequacy	3.97	4.2	−0.23
5	Space allocation adequacy	4	4.13	−0.13
6	Energy goal management	4	4.2	−0.2
7	Safety management	4.43	4.47	−0.04
8	Manpower procurement	3.9	4.36	−0.46
Overall mean		4.02	4.31	−0.29

## Discussion and Conclusion

University facilities should support the provision of academic and rest functions through proper management of these facilities. This enables students to perform various activities such as study and rest. However, as mentioned above, the current domestic UFM does not adequately reflect the needs of the facility users (students). It is mainly focused on old-fashioned methods, such as reactive maintenance, where the problems are dealt with when they occur. This happens due to the lack of effective FM strategies for university facilities. Based on the satisfaction and importance data of the current facilities management items, it is surely necessary to prepare an effective university facilities management plan. Based on the results of this research, the following conclusions are reached.

First, through the extensive review of the literature and two rounds of Delphi survey, 35 UFM items and 8 UFM strategies have been extracted. Those items and strategies are used in the survey for the main users of university facilities.

Second, in order to find out which UFM items need more focus on, IPA is conducted. The answers on the importance and the performance of each factor are graphically displayed on the two-dimensional grid. The priority of management is determined by where each factor is located on the grid. Respondents are asked about the questions such as “How important is this feature with respect to other features? How well did the feature perform?”

Third, in exchange for identify UFM items that affect the strategy of UFM, multiple linear regression analysis is conducted to examine the effect of the importance value on the UFM items on the importance value on the UFM strategies.

Fourth, after figuring out the difference in the importance of users and facility managers who actually run university facility, the average values of importance for the strategy of UFM of two groups are compared and analyzed.

As a result of the four stages listed above, this research attempts to provide basic data on preparing the UFM plan. Therefore, it is possible to apply the method that reflects the needs of actual users in preparing future UFM plans throughout the research methods proposed in this research. Since UFM is unique from the general FM for other types, the research focuses on university users’ opinions and utilizes expert’s opinions through the Delphi survey to show the direction how the UFM should be heading to the future.

However, since this research is conducted on the limited spatial scope of university facilities, it was not able to cover the spatial scope of the entire university campus where students stay, such as dormitories and student restaurants (places that affect the overall quality of university education). Further research on the supporting facilities is required in the future. Therefore, in-depth research should be conducted on all facilities on university campuses to further investigate users’ satisfaction, importance factors, and differences in perceptions of facility managers to prepare comprehensive UFM plans. Through such supplementary work, it is highly suggested to prepare a UFM plan that can cover the spatial scope of the entire university. As a result, it will be possible to establish an effective university management plan that can maximize the satisfaction of the main users, students.

## Data Availability Statement

The datasets generated for this study are available on request to the corresponding author.

## Author Contributions

MK conducted the related background study, collected the data, and analyzed them. JK designed and directed the entire research.

## Conflict of Interest

The authors declare that the research was conducted in the absence of any commercial or financial relationships that could be construed as a potential conflict of interest.

## References

[B1] Akinyode B. (2014). Students’ satisfaction and perception on rented apartments in nigeria: experiment of lautech students. *Int. J. Bus. Soc. Res.* 5 58–70.

[B2] Appa (2007). *Thought Leaders Series. Educational Facilities and the Impact of Technology, Expectations, and Competition.* Alexandria: APPA.

[B3] Astin A. (1993). Diversity and multiculturalism on the campus. *Change.* 25 44–52.

[B4] Cho C. Lee H. (2008). A study on the privatization of school facilities maintenance. *J. Korean Inst. Educ. Facil.* 15 39–49.

[B5] Hassanain M. (2008). On the performance evaluation of sustainable student housing facilities. *J. Facil. Manag.* 6 212–225. 10.1108/14725960810885989

[B6] Jeon S. (2009). *A Study on the Classification System of University Facilities and Space.* Master’s Thesis, Kongju University, Gongju.

[B7] Kim C. (2016). *A Study on the Evaluation Index and Management Model of University Facilities.* Thesis, Chunnam National University, Yeosu.

[B8] Kim C. Jeong Y. Jeong U. (2006). A study on the application of FMS for university campus facility demand management. *J. Korean Inst. Archit.* 22 125–134.

[B9] Kim M. (2019). *Facility Management Item Analysis for Efficiency Improvement of University Facility Management.* Master’s Thesis, Kyung Hee University, Seoul.

[B10] Kim Y. Kim M. Kim J. (2018). Development of key performance indicators for the improvement of university facility management services in Korea. *J. Asian Archit. Build. Eng.* 17 313–320. 10.3130/jaabe.17.313

[B11] LeBlanc G. Nguyen N. (1999). Listening to the customer’s voice: examining perceived service value among business college students. *Int. J. Educ. Manag.* 13 187–198. 10.1108/09513549910278106

[B12] Lee H. Cho C. (2008). A study on the efficiency management of national university facilities and spaces. *J. Korean Inst. Educ Facil. Assoc.* 15 21–32.

[B13] Martilla H. James J. (1977). Importance-performance analysis. *J. Mark.* 41 77–79.

[B14] Reynolds G. Cain D. (2006). *Final Report on the Impact of Facilities on the Recruitment and Retention of Students.* Alexandria: APPA Center for Facilities Research.

[B15] Ruben B. D. (1995). *Quality in Higher Education.* New Brunswick: Transaction Publishers.

[B16] Ryu S. Lee H. (2008). A study on development of the assessment category and items for university facility management. *J. Korean Inst. Educ. Facil.* 15 22–29.

[B17] Shin E. Kim J. (2012). A comparative analysis of domestic and overseas university facility management for green campus. *J. Korean Educ. Facil. Assoc.* 9 45–53. 10.7859/kief.2012.19.1.045

[B18] Shin E. Kim Y. Kim J. (2015). Direction of FM service development for university facilities based on KS facility management standards. *Korean Inst. Educ. Facil.* 22 37–45.

[B19] Shon B. Kim J. Kim S. (2003). A study on developing standardized index of evaluation for upgrading the school facilities. *J. Korean Inst. Educ. Facil.* 10 23–34.

[B20] Yorke M. Bridges P. Woolf H. (2000). Mark distributions and marking practices in UK higher education: some challenging issues. *Act. Learn. Higher Educ.* 1 7–27. 10.1177/1469787400001001002

[B21] You S. Lee H. (2008). Development of classification system and evaluation items for university facility management. *J Korean Inst. Educ. Facil. Assoc.* 15 22–29.

[B22] Yun S. Park J. Jeong S. (2009). A study on the effective asset management business model for campus facilities. *J. Archit. Inst. Korea Struc. Constr.* 25 139–146.

